# RF-to-DC Characteristics of Direct Irradiated On-Chip Gallium Arsenide Schottky Diode and Antenna for Application in Proximity Communication System

**DOI:** 10.3390/s140203493

**Published:** 2014-02-20

**Authors:** Farahiyah Mustafa, Abdul Manaf Hashim

**Affiliations:** 1 Faculty of Electrical Engineering, Universiti Teknologi Malaysia, Skudai, Johor 81310, Malaysia; E-Mail: farzies@gmail.com; 2 Malaysia-Japan International Institute of Technology, Universiti Teknologi Malaysia, Jalan Semarak, Kuala Lumpur 54100, Malaysia; 3 MIMOS Berhad, Technology Park Malaysia, Kuala Lumpur 57000, Malaysia

**Keywords:** direct integration, Schottky diode, dipole antenna, proximity communication

## Abstract

We report the RF-to-DC characteristics of the integrated AlGaAs/GaAs Schottky diode and antenna under the direct injection and irradiation condition. The conversion efficiency up to 80% under direct injection of 1 GHz signal to the diode was achieved. It was found that the reduction of series resistance and parallel connection of diode and load tend to lead to the improvement of RF-to-DC conversion efficiency. Under direct irradiation from antenna-to-antenna method, the output voltage of 35 mV was still obtainable for the distance of 8 cm between both antennas in spite of large mismatch in the resonant frequency between the diode and the connected antenna. Higher output voltage in volt range is expected to be achievable for the well-matching condition. The proposed on-chip AlGaAs/GaAs HEMT Schottky diode and antenna seems to be a promising candidate to be used for application in proximity communication system as a wireless low power source as well as a highly sensitive RF detector.

## 1. Introduction

In order to increase the performance of silicon (Si)-based ultra-large-scale-integrated circuits (ULSIs), miniaturization of complementary metal-oxide-semiconductor (CMOS) transistors is needed [1]. However, further miniaturization seems to be difficult due to the increase in gate leakage current [2], short channel effects [3], *etc.*, although several innovations such as strained Si [4], high-*k* materials [5] and tri-gate [6] structure have been introduced. The most promising breakthrough to further increase the performance of ULSIs is by introducing new channel materials with higher carrier mobilities than Si, such as gallium arsenide (GaAs) [7,8]. Therefore, co-integration of GaAs on Si should lead to the realization of the so-called advanced heterogeneous integration on a Si platform [9], where this material is not only used for the fabrication of high speed transistor, but also for the fabrication of other functional devices such as on-chip low power sources [10], sensors [11,12], optical devices [13], detectors [14–16] and solar batteries [17]. Nowadays, there is extensive research on the growth of GaAs on Si [18–20], which has seemed to accelerate the realization of such technology. One of the potential GaAs based device structure to be integrated on Si is a rectenna device which can provide dual functions as wireless low power source and RF power detector [15]. An on-chip rectenna device is defined as a combination of an on-chip Schottky diode and a planar antenna.

Since the 1970s, one of the major reasons for intensive research on rectennas has been due to the development of solar power satellites in space for energy harvesting from sunlight [21]. In recent years, interest has turned to the exploitation of on-chip rectennas as wireless low power sources for application in wireless microelectronic systems. The most common application of rectennas is in radio frequency identification (RFID) tags [22], proximity cards and contactless smart cards [23], which contain an integrated circuit (IC) which is powered by a small rectenna element. When the device is brought near to an electronic reader unit, radio waves from the reader are received by the rectenna, powering up the IC, which transmits its data back to the reader.

In 2002, Suh *et al.* [24] presented a rectenna designed for over 100 milliwatt (mW) rectification and whose RF-to-DC power conversion efficiency was less than 20% at the 1 mW microwave input. Tu *et al.* [25] published an experimental work on a 5.8 GHz rectenna using a dipole antenna with a conversion efficiency of 76% at a load resistance of 250 Ω. In 2011, Harouni *et al.* [26] reported a 2.45 GHz rectenna with maximum conversion efficiency of 63% at a load resistance of 1.6 kΩ. These reports have thoroughly discussed the results of integrated large-scale discrete diodes and antennas with the insertion of the matching circuits [24–29]. Consequently, due to the large dimensions, these concepts are not suitable for several tens of millimeter-scale on-chip systems. Thus, on-chip rectenna devices of small dimensions with the omission of impedance matching circuit need to be developed for their application in on-chip proximity communication systems.

Recently, we reported the design, fabrication and characterization of individual n-AlGaAs/GaAs high-electron-mobility-transistor (HEMT) Schottky diodes [15] and planar antennas [30,31] in order to understand the feasibility of direct integration of both components. Direct injection of RF signals from a signal source were found to be well detected and rectified by the fabricated Schottky diodes which possessed cut-off frequencies of up to several tens of GHz, and a stable DC output voltage was generated. High RF-to-DC conversion efficiency of up to 50% was obtained with series connection between the diode and the load [15].

In this paper, we report the RF-to-DC characteristics of a Schottky diode where the diode and load are connected in parallel under direct injection of the RF signal. The rectifying characteristics of the Schottky diode where the signal is irradiated from different transmitting dipole antennas to the integrated dipole antenna are also reported. This experiment was conducted in order to understand the performance of the integrated devices for real practical applications. The results show the potential breakthrough for direct on-chip integration towards realization of low power rectenna devices for their advanced heterogeneous integration on a Si platform.

## 2. Fabrication of the Integrated Device

An *n*-AlGaAs/GaAs HEMT structure has been chosen as a substrate. This structure is capable of providing higher electron mobility due to its two-dimensional electron gas (2DEG) layer defined at the interface of the *n*-doped AlGaAs layer and undoped GaAs layer. Therefore, the *n*-AlGaAs/GaAs HEMT structure is promising for the fabrication of high-speed and high-frequency devices. Co-integration of various kinds of functional devices including rectenna devices on the same core material structure is more practical in terms of fabrication processes and cost. Thus, the development of rectenna devices based on such a structure has been considered in this study.

**Figure 1.**
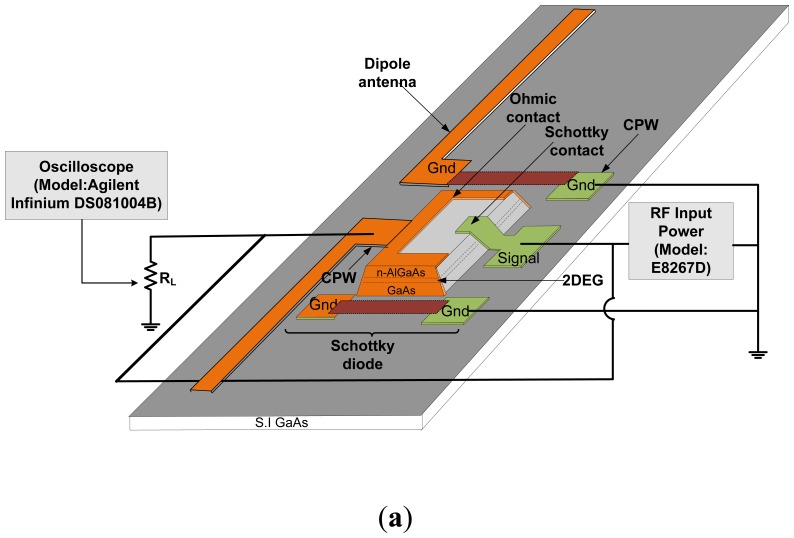

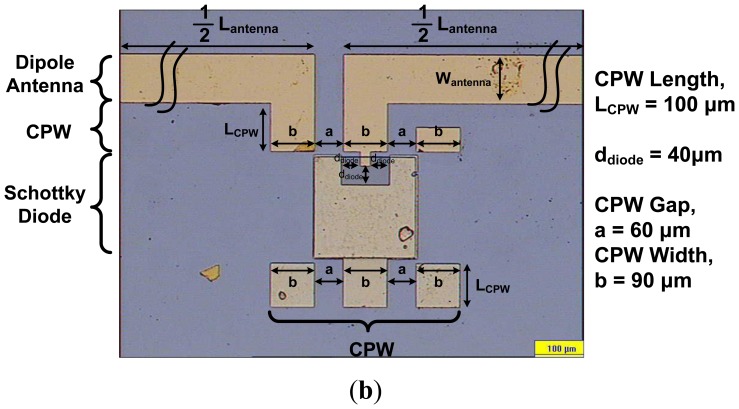
(**a**) Schematic and (**b**) top view photo of the rectenna device.

In this work, we fabricated the CPW and dipole antenna structure on the semi-insulated (SI) GaAs layer, and not directly on the n-type HEMT structure, as shown in Figure 1a. The HEMT structures was etched to the SI layer during the process of mesa formation by using a mixture of sulphuric acid, H_2_SO_4_, hydrogen peroxide (H_2_O_2_) and deionized (DI) water at 25 °C for 18 s. Formation of CPW and the dipole antenna on a SI layer seems to reduce the RF losses as the signal is travelling through the CPW. The details of the materials and the fabrication processes have been described in [10,15,31]. Figure 1b shows the top-view photo of the rectenna device. Table 1 summarizes the device dimensions and the operating frequencies. The CPW structure was designed so that it produces the characteristic impedance, *Z_0_*, of 50 Ω. This CPW structure also permits direct injection of the RF signal through a Cascade GSG Infinity-150 microprober.

## 3. Result and Discussion

### 3.1. RF Characteristics and Conversion Efficiency of the Schottky Diode and Dipole Antenna by Direct Signal Injection

**Figure 2.**
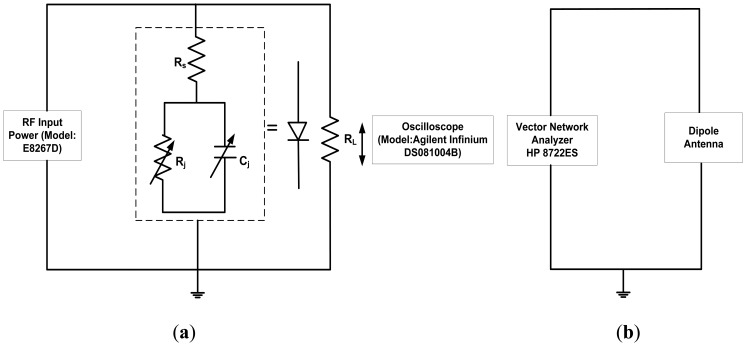
The circuit configuration for: (**a**) the Schottky diode and (**b**) the dipole antenna in direct injection experiment.

In this study, a RF direct injection measurement was conducted in order to confirm several parameters such as: (1) the input power needed to turn on the diode; (2) the maximum input power generated by the signal generator; (3) the operating frequencies of the fabricated Schottky diode; (4) the resonant frequency of antenna; and (5) the RF characteristics of the Schottky diode. Figure 2a,b shows the circuit configuration of the direct injection experiment for the Schottky diode and dipole antenna, respectively. As shown in Figure 2a, the RF signals were directly injected at the input side of diode using a microprober. The load resistance, *R_L_* of 50 Ω was connected to the diode in parallel and grounded to the RF source. When the injected voltage is equal or larger than threshold voltage of diode, the diode will be turned on. The generated DC voltage across the diode which also defined as an output voltage is measured at the connected load using an oscilloscope. The output voltage increases with the increase of injected voltage. From this measurement, the turn on voltage, the operating frequencies and the RF characteristics of the diode were evaluated. Next, an HP8722ES Network Analyzer (VNA) equipped with the same microprober, as shown in Figure 2b, was used to measure and confirm the resonant frequency of the dipole antenna.

**Figure 3.**
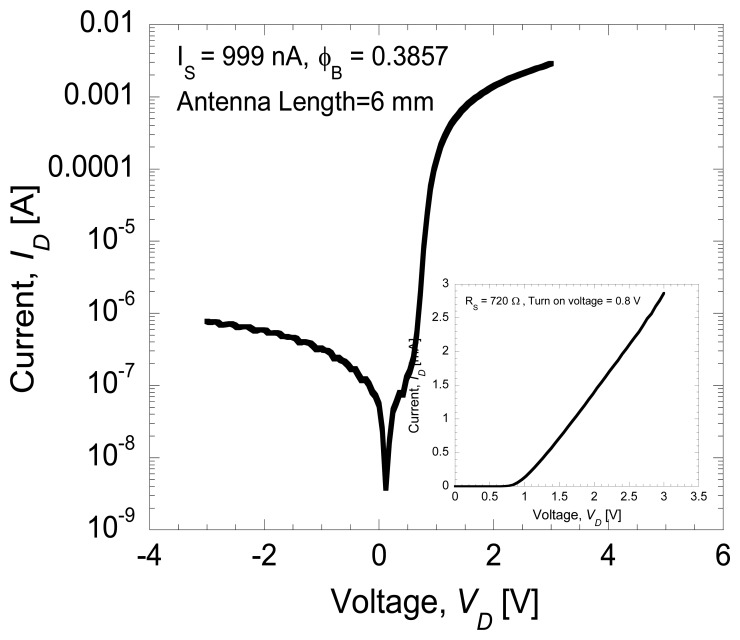
DC *I-V* characteristics of the fabricated on-chip Schottky diode.

Figure 3 shows the DC *I-V* curve of the Schottky diode with series resistance of 720 Ω defined at a slope between 2 and 3 V. The threshold voltage was estimated to be 0.8 V as shown in the inset of Figure 3. The reverse leakage current for the fabricated device was 999 nA and the Schottky barrier height (SBH) was calculated to be 0.3857 eV. This calculated experimental barrier height is lower than the theoretical calculated value of 1.443 eV. The discrepancy of Schottky barrier height values was discussed in [15,31].

**Figure 4.**
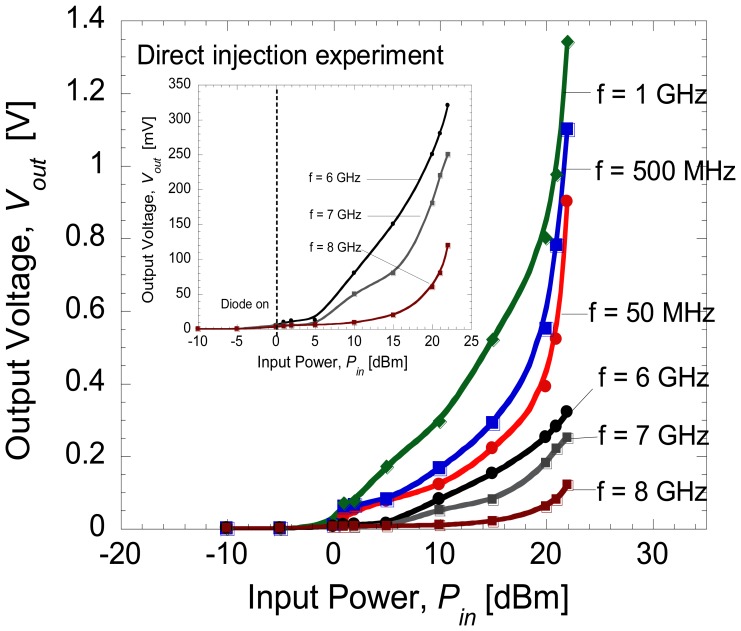
Rectified output voltages as a function of input voltages at different frequency level.

**Figure 5.**
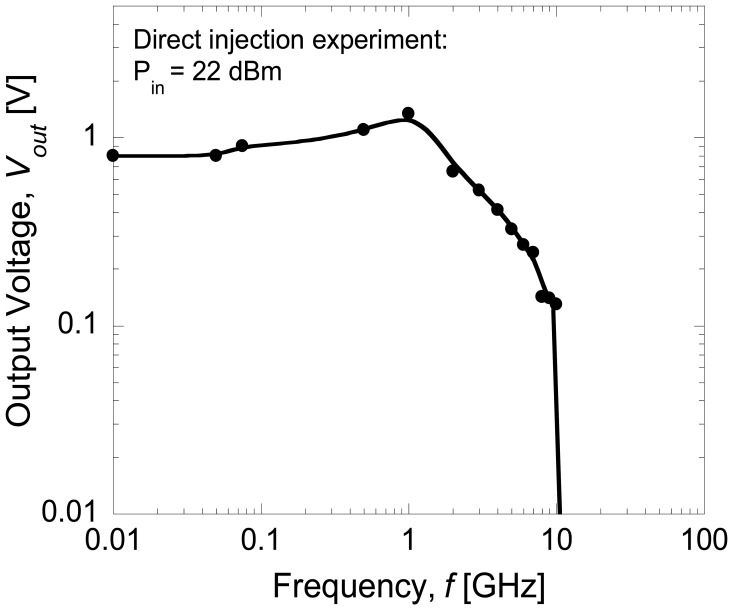
Rectified output voltages as a function of the frequencies at input power of 22 dBm.

Figure 4 shows the average rectified voltages, *V_out_* as a function of input power, *P_in_* at different frequency levels. In this study, the turn-on voltage of the Schottky diode is estimated to be around 0.8 V as shown in the inset of Figure 3. Therefore, an input power of more than 0 dBm (=0.8 V) must be applied in order to turn the diode on. Furthermore, the output voltage of around 1.4 V measured at the load is the maximum DC output voltage obtainable across the Schottky diode at an input power of 22 dBm (=2 V). As expected, the output voltage increases with the increase of injected voltage. Here, the difference between the input voltage and output voltage is around 0.6 V, which is attributed to the loss. Also shown in Figure 4, it is noticed that the maximum input power that can be injected is 22 dBm due to the limitation of the signal generator. Figure 5 shows the rectified output voltages as a function of frequency at maximum input power of 22 dBm. As shown in Figure 5, the diode shows the maximum output voltage at 1 GHz and the cut-off frequency is 10 GHz.

**Figure 6.**
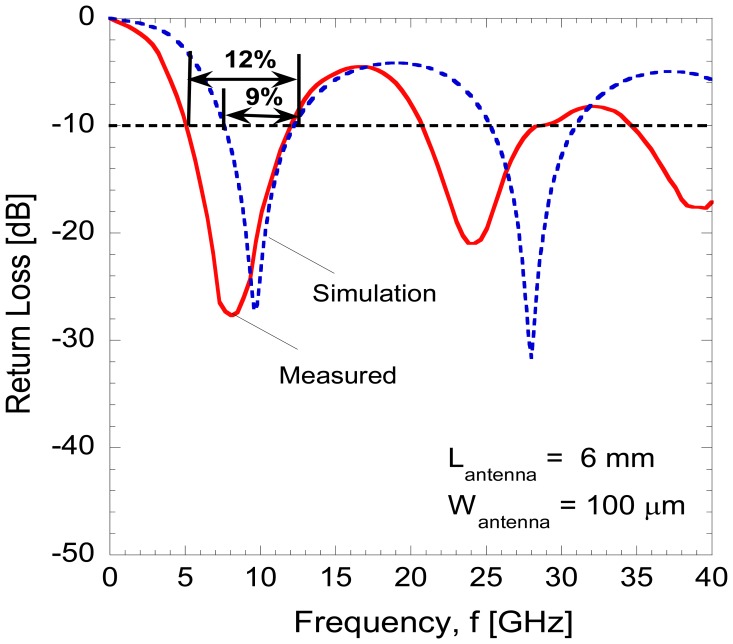
Measured and simulated return loss of the dipole antenna.

Figure 6 shows the return loss characteristics as a function of frequency for the fabricated antenna. The dipole antennas have also been designed and simulated using the commercial Electromagnetic Sonnet Suites simulator. As shown in Figure 6, there was almost 3% difference of frequency bandwidth at −10 dB between the measured and simulated response for the first resonant harmonic. Such a small discrepancy is commonly observed [32] due to a variation in parameters such as the loss tangent for the fabricated device, whereas a simulator is dealing with an ideal parameter. It can also be clearly seen that a high return loss magnitude down to −28 dB at 7 GHz was obtained experimentally. This concludes that the resonant frequency for the antenna is 7 GHz and it is still in the range of the operating frequency of the integrated diode.

Using Equation (1), the RF-to-DC conversion efficiency of the fabricated Schottky diode at several frequencies was calculated [33]:

(1) $$\eta =\frac{P_{\text{out}}}{P_{in}}x\;100\%$$

**Figure 7.**
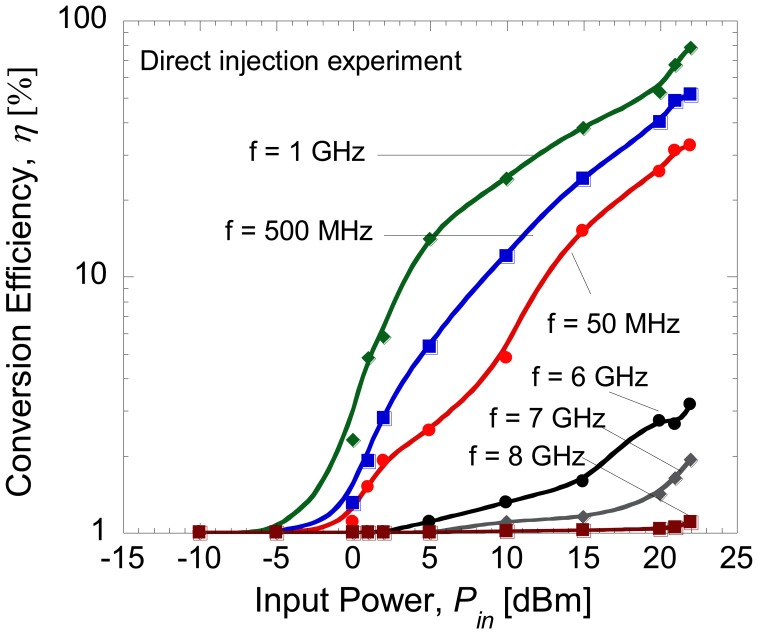
Conversion efficiency as a function of input power at several frequencies.

Here, *P_out_* is the DC power produced at the load resistance, *R_L_* and *P_in_* is the injected power at the input side of diode. Figure 7 shows the measured conversion efficiency of the diode as a function of input power at different frequencies. Here, it can be seen that up to 80% conversion efficiency was obtained at frequency of 1 GHz which has been considered as the most optimum operating frequency of the fabricated diode. The rectification by direct injection should give the maximum conversion efficiency that is obtainable in the fabricated diode due to its minimal loss. From our previous study on individual diodes presented in [15], only 50% of the RF-DC conversion efficiency was obtained with a serial configuration of the diode and load. It is noted that the total resistance of the diode presented in [15] was 1.37 kΩ. Therefore, this seems to suggest that these two conditions: (1) reducing the total series resistance down to several Ω and (2) applying a parallel connection of diode and load may lead to improvement of the RF-to-DC conversion efficiency. As shown in Figure 2a, the diode is also modeled with a junction capacitance element, *C_j_*. This capacitance determines the cut-off frequency of the diode as described in [31]. Therefore, both configurations of diode and load, *i.e.*, series and parallel connection, should be able to generate RF-to-DC conversion characteristics within the range of the operating frequency of diode. As reported in [31], an additional external capacitor may be used to improve the stability of the DC output voltage. In this parallel connection, the built-in internal capacitor of the oscilloscope has been confirmed to be sufficient in producing stable DC output voltages. McSpadden *et al.* [29] also reported a high RF-to-DC conversion efficiency of 82% using a similar parallel connection of diode (5.8 GHz) and load (327 Ω).

### 3.2. Rectifying Characteristics of the Integrated Device by Direct Irradiation from Antenna-to-Antenna

**Figure 8.**
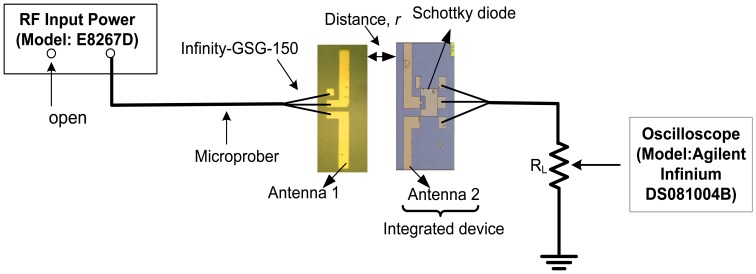
Measurement configuration for direct irradiation from antenna-to-antenna.

Figure 8 shows the measurement configuration for the irradiation by the antenna-to-antenna method. The irradiation was performed using a similar dipole antenna structure (denoted as antenna 1) which is used to transmit the signal to the receiving antenna of the integrated device (denoted as antenna 2). In this experiment, a signal generator is used to supply an RF signal to antenna 1. In order to turn on the diode (turn on voltage =0.8 V), sufficient power of more than 0 dBm should be received by antenna 2. As shown in Figure 7, the resonant frequency of fabricated dipole antenna with length of 6 mm was ∼7 GHz and it was in the range of operating frequencies of diode (10 MHz to 10 GHz). Therefore, the rectifying operation should be feasible. The diode and load (*R_L_* = 50 Ω) were connected in parallel configuration and the load was grounded to the RF source.

**Figure 9.**
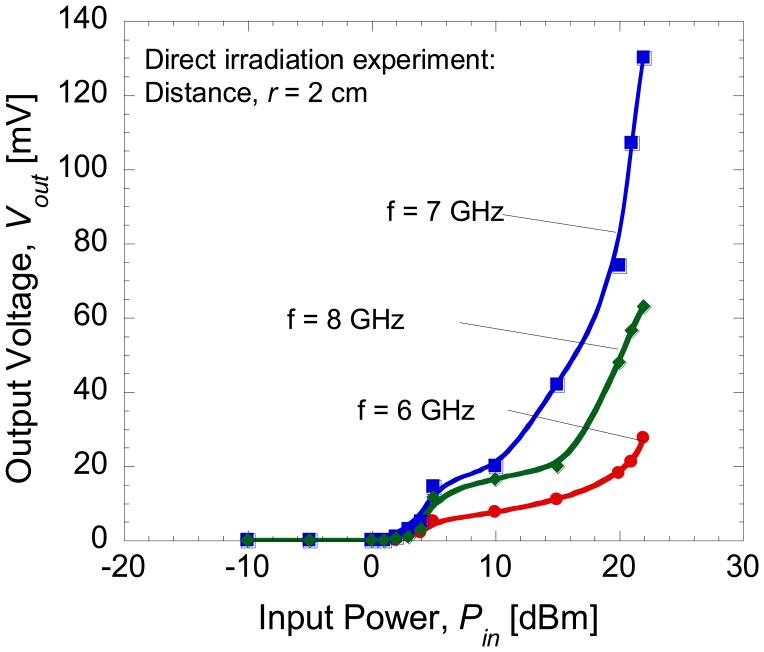
Rectified output voltages as a function of input power at distance of 2 cm.

**Figure 10.**
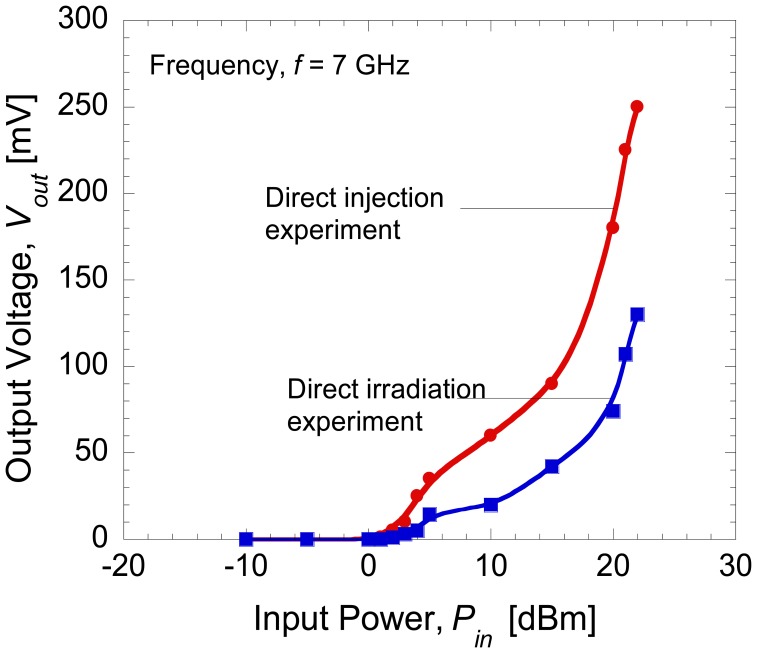
Rectified output voltage at a frequency of 7 GHz.

Figure 9 shows the rectified output voltages when the distance, *r* between antenna 1 and antenna 2 was set at 2 cm. The maximum output voltage around 130 mV was generated at the load for a frequency of 7 GHz since the resonant frequency of the antenna was 7 GHz. Figure 10 shows the comparison of the rectified output voltages for the case of direct injection and RF irradiation at a frequency of 7 GHz. It can be seen that only half of the output voltage was produced by the integrated devices for the case of direct irradiation. Higher output voltage of up to volt (V) range is expected to be achievable if the resonant frequency of dipole antenna is well matched to the optimum operating frequency of diode that produces maximum rectified output. This seems to suggest that such a purpose can be achieved by replacing the present dipole antenna which is a narrow bandwidth type with an antenna that has wider bandwidth and high return loss so that such demerits can be eliminated. This is because the optimum frequency of diode which produces maximum rectified output is not controllable even though the ranges of its operating frequencies are predictable. The optimization of antenna structure is more easy and a preferable direction in order to realize maximum conversion efficiency.

**Figure 11.**
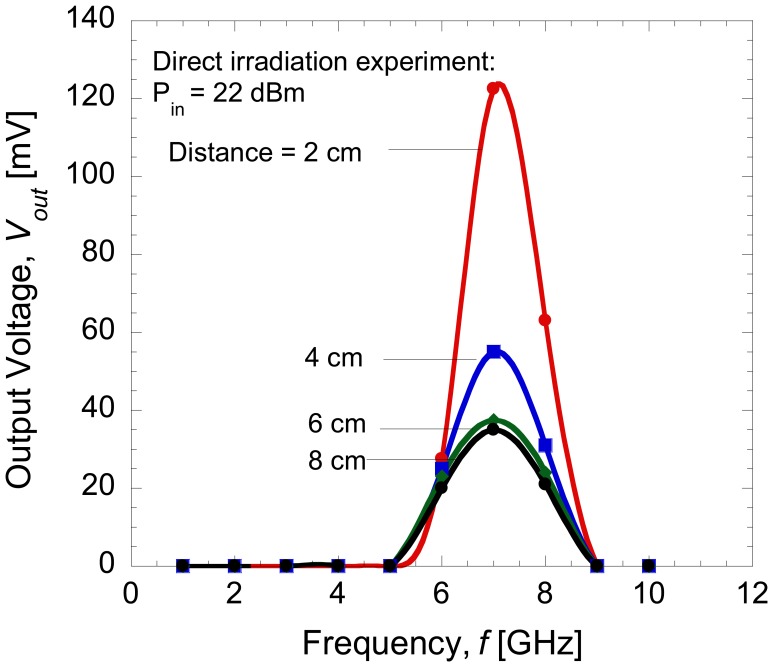
Rectified output voltage at input power of 22 dBm with different distance.

Finally, the dependence of the distance, *r* on the output voltage was evaluated. The distance could only be varied from 2 to 8 cm due to the space limitations of the measurement setup. Figure 11 shows the rectified output voltage as a function of frequency at an input power of 22 dBm and different distances.

Here, it can be clearly seen that a maximum rectified output voltages were obtained at 7 GHz and a maximum voltage of 35 mV was still obtainable for the distance of 8 cm. From these presented results, the proposed on-chip AlGaAs/GaAs HEMT Schottky diode and antenna seem to be promising candidates to be used for application in proximity communication systems as a wireless low power source as well as a highly sensitive RF detector device.

## 4. Conclusions

In conclusion, the rectification by the integrated Schottky diode and dipole antenna via CPW transmission line under direct irradiation from antenna-to-antenna was achieved without insertion of any matching circuit. Higher output voltages, up to the volt range, are expected to be achievable if the resonant frequency of the dipole antenna is well matched to the optimum operating frequency of the diode that produces the maximum rectified output. Despite of the large mismatch in the frequency between the diode and antenna, output voltages of several tens of mV were still obtainable for a distance of 8 cm. This seems to suggest the feasibility of using such integrated device structures in proximity communication systems.

## Figures and Tables

**Table 1.** Device dimensions and operating frequencies of the Schottky diode and antenna.

**Schottky Diode**		**Antenna**	
Distance, *d*	40 μm	*L_antenna_*	6 mm
Area, *A*	20 μm × 20 μm	*W_antenna_*	100 μm
Working frequency	10 MHz–10 GHz	Resonant frequency	7 GHz
